# Extraction and Separation of Volatile and Fixed Oils from Berries of *Laurus nobilis* L. by Supercritical CO_2_

**DOI:** 10.3390/molecules13081702

**Published:** 2008-08-13

**Authors:** Hanen Marzouki, Alessandra Piras, Bruno Marongiu, Antonella Rosa, M. Assunta Dessì

**Affiliations:** 1 Laboratoire de Botanique et de Biologie végétale, Université de Tunis, Tunisia; E-mail: hanen_marzouki7@yahoo.fr; 2 Dipartimento di Scienze Chimiche, Università degli Studi di Cagliari, Cittadella Universitaria di Monserrato, SS 554, Km 4.500, 09042 Cagliari, Italy; E-mail: apiras@unica.it; 3 Dipartimento di Biologia Sperimentale, Sezione di Patologia Sperimentale, Università degli Studi di Cagliari, Cittadella Universitaria di Monserrato, SS 554, Km 4.500, 09042 Cagliari, Italy; E-mails: anrosa@unica.it; dessima@unica.it

**Keywords:** *Laurus nobilis* L., essential oil, fixed oil, fatty acids, supercritical extraction, carbon dioxide

## Abstract

Isolation of volatile and fixed oils from dried berries of *Laurus nobilis* L. from Tunisia have been obtained by supercritical fractioned extraction with carbon dioxide. Extraction experiments were carried out at a temperature of 40 °C and pressures of 90 and 250 bar. The extraction step performed at 90 bar produced a volatile fraction mainly composed of (*E*)-β-ocimene (20.9%), 1,8-cineole (8.8%), α-pinene (8.0%), β-longipinene (7.1%), linalool acetate (4.5%), cadinene (4.7%), β-pinene (4.2%), α-terpinyl acetate (3.8%) and α-bulnesene (3.5%). The oil yield in this step of the process was 0.9 % by weight charged. The last extraction step at 250 bar produced an odorless liquid fraction, in which a very small percentage of fragrance compounds was found, whereas triacylglycerols were dominant. The yield of this step was 15.0 % by weight. The most represented fatty acids of the whole berry fixed oil were 12:0 (27.6%), 18:1 n-9 (27.1%), 18:2 n-6 (21.4%), and 16:0 (17,1%), with the 18:1 n-9 and 18:2 n-6 unsaturated fatty acids in particular averaging 329 μg/mg of oil.

## Introduction

The Lauraceae comprise 32 genera and about 2,000-2,500 species. *Laurus nobilis* L., (bay) a member of the family named Apollo’s Laurel in mythology, is a plant native to the southern Mediterranean region and widely cultivated mainly in Europe and the USA as an ornamental plant [1]. Bay is a plant of industrial importance, used in foods, drugs, and cosmetics. The dried leaves and essential oils are used extensively in the food industry for seasoning of meat products, soups and fishes. Its antimicrobial and insecticidal activities are other factors for which bay is used in the food industry as a food preservative. The essential oil is also used as a folk medicine, especially for the treatment of rheumatism and dermatitis [2]. Although several isolation and biological activity studies have been carried out on the leaves of *L. nobilis*, there has been very little work on its fruits. Laurel berries are one-seeded ovoid fruits with a dark purple, thin, brittle, wrinkled pericarp, which when broken discloses the seed kernel, the seed-coats adhering to the inner surface of the pericarp. The fruits contain both fixed and volatile oils, which are mainly used in soap making [3]. The oil extracted from berries contain fatty acids (lauric, 54%; palmitic, 5%; oleic, 15%; and linoleic, 17%) [4] and volatile compounds such as β-ocimene (22%), 1,8-cineole (9.5%), bicyclogermacrene (4.5%) and β-elemene (2%) [2]. 

In recent years supercritical fluid extraction (SFE) has received increased attention as an important alternative to conventional separation methods. Indeed, it has been demonstrated that SFE can produce superior quality products characterized by the absence of artifacts and by a better reproduction of the original flavour or fragrance. Supercritical fluids have adjustable extraction characteristics, due to their density, which can be controlled by changing the pressure or temperature. At lower pressures (near the critical point) volatile components, such as essential oils, are selectively extracted, while other components present in the vegetable matter such as waxes, resins and dyes have low solubility under these conditions. Globally, previous results [5,6,7] show that at extraction temperatures between 40 and 50°C and at extraction pressures lower than 100 bar, higher molecular-weight compounds are not coextracted with essential oils. A subsequent further run at higher pressure (250 bar) using the same exhausted matrix can been performed in order to obtain the fixed oil.

Vegetable oils from seeds are traditionally produced by hexane extraction from ground seeds. The process is very efficient, but hexane elimination after extraction is a major problem. Three distillation units in series, operated under vacuum and other ancillary apparatus (deodorizers, degummers, etc.), have to be used. The possible thermal degradation of the oil and the incomplete hexane elimination are the main drawbacks of this process. Consequently, several authors have proposed the substitution of the traditional process by Supercritical CO_2_ (SC-CO_2_) extraction of oil from seeds. Indeed, triglycerides forming seed oils are readily soluble in SC-CO_2_ at 40 °C and at pressures higher than about 250 bar. After extraction, the SC-CO_2_ tryglicerides solution is sent to a separator working at subcritical conditions. This operation reduces to near zero the solvent power of CO_2_ and allows the recovery of oil. The complete elimination of gaseous CO_2_ from oil is also obtained in the separator. The SFE of several seed oils has been successfully performed up to the pilot scale [8].

In this paper, we continue with our studies on the extraction and characterization of the SFE extracts derived from *L. nobilis* widespread in Mediterranean countries [9,10]. The objective of this work was to explore the potential application of supercritical CO_2_ to the extraction of volatile and fixed oils from *L. nobilis* berries growing in Tunisia.

## Results and Discussion

### Volatile oil

The volatile oil of *L. nobilis* berries was obtained by SFE at 90 bar and 40 °C (CO_2_ density, ρ_CO2_ = 0.479 g cm^-3^) and the yield, expressed as the percentage by weight of the oil with respect to the weight of the material charged in the extractor, was 0.9%. The essential oil extracted using the supercritical technique was compared with the oil extracted by hydrodistillation (HD) performed on the same starting material (yield of 0.8 %). 

The essential oils were analyzed by GC/MS to monitor their composition. Table 1 gives the contents of the supercritical extracts and the essential oil obtained by hydrodistillation. Chemical analysis revealed that essential oil extracted under SFE conditions had a high content of (*E*)-β-ocimene (20.9%), 1,8-cineole (8.8%), α-pinene (8.0%), β-longipinene (7.1%), δ-cadinene (4.7%), linalyl acetate (4.5%), β-pinene (4.2%), α-terpinyl acetate (3.8%) and α-bulnesene (3.5%). The compounds isolated by HD were practically the same as those extracted by SFE. Although, the HD oil contains a little bit more monoterpenes and the SFE extract had a higher content of sesquiterpenes. The main differences observed were the content of linalool, which was greater in HD oil (4.2%) than in SFE extract (2.2%), and the content of linalyl acetate, which showed an opposite trend, 1.3% in HD oil and 4.5% in SFE extract. This behaviour is due to hydrolytic transformation of linalyl acetate to linalol in hydrodistillation process.

The essential oil content shows variations in plants of different geographical origin and also in different part of the tree. Recentely, Yalçin *et al.* studied the composition of *L. nobilis* oil collected from the Northern Cyprus Montains (Turkey). They reported that the essential oil of leaves is characterized by a high content of 1,8-cineole (58.59%), terpinen-4-ol (4.25%), α-pinene (3.39%), sabinene (3.32%) and β-pinene (3.25%) [11]. In our previous studies on the chemistry of Tunisian *L. nobilis* [10,12], considerable differences were observed in the essential oil composition between stems, leaves, buds and flowers. The essential oil of the different plant organs contained the same compounds, but the quantitative differences between all main compounds were quite large. Thus, 1,8-cineole, α-terpinyl acetate, methyl eugenol, eugenol and linalool, which are the basic components of the essential oil of leaves, buds and flowers, were present in small quantity in the fruits. On the other hand, (*E*)-β-ocimene is not present in stems, leaves, buds and flowers but it is a major component in the fruit oil (21-24%). Similar results were obtained by Kilic *et al.* for the essential oil of Turkish bay: the volatile compounds in bay fruits mainly consisted of 1,8-cineole (9.5%) and (*E*)-β-ocimene (22.1%), which is not present in leaves [2]. Also, Castilho *et al.*, found (*E*)-β-ocimene and germacrene D predominating in the essential oil extracted from fruits of Portuguese *L. nobilis* [13]. On the other hand, Hafizoglu *et al.* studied the composition of *L. nobilis* from Turkey and they reported 4-terpineol to be the main component in the fruit essential oil [4].

**Table 1:** Retention times, T_r_, retention indices, I_R_, and chromatographic area percentages of constituents of laurel essential oil obtained from berries by SFE and by HD.

T_r_	I_R_	Compound	SFE	HD	Identification
5.37	933	α-pinene	8.0	10.3	MS, I_R_, Inj
5.74	948	camphene	2.6	3.8	MS, I_R_
6.37	973	sabinene	1.8	2.6	MS, I_R_, Inj
6.48	977	β-pinene	4.2	5.8	MS, I_R_, Inj
8.12	1031	1,8-cineole	8.8	8.1	MS, I_R_, Inj
8.69	1047	(*Z*)*-*β-ocimene	2.0	3.0	MS, I_R_
8.80	1050	(*E*)-β-ocimene	20.9	23.7	MS, I_R_
10.56	1101	linalool	2.2	4.2	MS, I_R_, Inj
13.14	1166	para-mentha-1,5-dien-8-ol	1.5	1.5	MS, I_R_
16.87	1256	linalyl acetate	4.5	1.3	MS, I_R_
18.11	1286	bornyl acetate	2.9	2.1	MS, I_R_, Inj
20.78	1350	α-terpinyl acetate	3.8	3.0	MS, I_R_, Inj
22.44	1390	β-cubebene	2.2	1.9	MS, I_R_
22.51	1392	β-longipinene	7.1	6.8	MS, I_R_
23.08	1405	methyl eugenol	1.4	1.0	MS, I_R_, Inj
23.60	1418	(*E*)-caryophyllene	2.5	1.9	MS, I_R_, Inj
26.07	1480	germacrene D	2.7	1.8	MS, I_R_
26.92	1501	viridiflorene	1.5	1.0	MS, I_R_
27.08	1505	α-bulnesene	3.5	2.7	MS, I_R_
27.39	1513	trans-cadinene	2.7	2.1	MS, I_R_
27.77	1523	δ-cadinene	4.7	3.9	MS, I_R_
29.81	1576	spathulenol	2.3	1.4	MS, I_R_
32.66	1652	α-cadinol	2.0	1.1	MS, I_R_
33.98	1688	5-isocedranol	2.1	1.1	MS, I_R_

**^a^**Identification has been realized by comparing mass spectra (MS), retention Indices (I_R_), and injection of authentic compound (Inj)

### Fixed oil

A further extraction at higher pressure (250 bar) and 40 °C (ρ_CO2_ = 0.886 g cm^-3^) was performed on the exhausted matrix for the extraction of fixed oil, with a yield of 15%. Quali-quantitative information on the individual fatty acids that compose the lipid classes of *L. nobilis* berries fixed oil was obtained by GC analysis. The composition of fatty acids present in the saponifiable matter of oil is shown in Table 2 and expressed as percentage of total fatty acids. The analyzed oil showed a concentration of approximately 48% of saturated fatty acids, 29% of monounsaturated, and 23% of polyunsaturated (saturated/unsaturated fatty acids ratio of 0.9). The most represented fatty acids of fixed oil from whole berry were 12:0 (27.6%), 18:1 *n-9* (27.1%), 18:2 *n-6* (21.4%), and 16:0 (17.1%). These results were within the range of fatty acids composition previously reported in literature for *L. nobilis* fat [13,14]. However the amounts of fatty acids greatly differed from those reported for daphne seed oil obtained by supercritical CO_2_ extraction [15].

Furthermore, the unsaturated fatty acids content in the oil was detected by HPLC (Table 3) as follows: 201.8 μg/mg of 18:1 *n-9*, 127.5 μg/mg of 18:2 *n-6*, and a minor amount of 18:3 *n-3* (5.5 μg/mg) and 16:1 *n-7* (1.9 μg/mg) (as mean values over six samples). The oxidation status of fatty acids was also measured by HPLC detection of conjugated diene fatty acid hydroperoxides (HP). The level of the oxidative products HP in the fixed oil was ca. 0.1 nmol/mg, comparable to that measured in olive oil [16], and prevalently derived from 18:2 *n-6* degradation, due to the great content of this fatty acid and the high stability of its hydroperoxides [17]. By HPLC, also the α-tocopherol level was measured as mean content of 166 ° 1.8 ng/mg of oil, comparable to that of olive oil [16].

**Table 2:** Fatty acids composition (%) of laurel fixed oil by GC.

Fatty acid	value	*sd*
10:0	0.4	*0.01*
12:0	27.7	*0.60*
14:0	1.0	*0.03*
16:0	17.1	*0.47*
16:1 *n-7*	0.3	*0.01*
18:0	1.5	*0.06*
18:1 *n-7*	0.9	*0.04*
18:1 *n-9*	27.2	*0.38*
18:2 *n-6*	21.5	*1.34*
18:3 *n-3*	1.2	*0.22*
20:0	0.2	*0.01*
20:1 *n-9*	0.7	*0.08*
		
SFA	47.8	*1.16*
MUFA	29.0	*0.35*
PUFA	22.7	*1.51*

SFA, saturated fatty acids. MUFA, monounsaturated fatty acids. PUFA, polyunsaturated fatty acids. Mean value and standard deviations of 6 samples

**Table 3:** Unsaturated fatty acids (UFA composition μg/mg weight) of laurel fixed oil by HPLC.

UFAs	value	*sd*
16:1 *n-7*	1.9	*0.01*
18:1 *n-9*	201.8	*1.81*
18:2 *n-6*	127.5	*1.11*
18:3 *n-3*	5.5	*0.24*

Data are mean values (6 samples) with standard deviations

## Conclusions

The overall results indicated that the extraction of laurel fruit oils can be successfully performed using supercritical CO_2_. The composition of oils obtained by this technique is largely influenced by solvent density. It has been confirmed that essential oil is selectively extracted only at low supercritical CO_2_ density; The essential oil was isolated by supercritical CO_2_ extraction coupled to a fractional separation technique: the waxes were recovered in the first separator and the the oil was recovered in the second one. Further, an additional extraction performed at higher pressure (250 bar) on the exhausted matrix allows the extraction of fixed oil (deprived of essential oil and waxes). Oils composition is comparable with the composition of the products obtained by conventional processes of extraction. 

## Experimental

### Chemicals

All solvents used, of the highest available purity, were purchased from Merck (Darmstadt, Germany). α-Pinene, β-pinene, 1,8-cineole, linalool, bornyl acetate, methyl eugenol, (*E*)-caryo-phyllene, fatty acids, fatty acids methyl esters and vitamin E (α-tocopherol) were obtained from Sigma, Aldrich or Fluka (Milan, Italy). Sabinene and α-terpinyl acetate were obtained from Extrasynthese (Genay, France). Desferal (deferoxamine methanesulfonate) was purchased from CIBA-Geigy (Basel, Switzerland). *cis,trans*-13-Hydroperoxyoctadecadienoic acid (c,t-13-HPODE) and *cis,trans*-9-hydro-peroxyoctadecadienoic acid (c,t-9-HPODE) were purchased from Cascade (Cascade Biochem. Ltd., London). All compounds were analytical standard grade. CO_2_ (99% purity) was supplied by Air Liquid Italia, Cagliari, Italy.

### Plant material

Mature fruits of *L. nobilis* (Lauraceae) were collected in November in El Agba, Tunisia, from cultivated plants The berries were air dried in the shade for 20 days. Before use the vegetable matter was ground with a Malavasi mill (Bologna, Italy) and the particles sizes were in the range (250–425) μm.

### Hydrodistillation

Hydrodistillation was performed for four hours in a circulatory Clevenger-type apparatus up to exhaustion of the oil contained in the matrix. About 100 g of material were charged. 

### SFE Apparatus

Supercritical CO_2_ extractions were performed in a laboratory apparatus, equipped with a 320 mL extraction vessel and two separator vessels of 300 and 200 mL, respectively, connected in series. Extraction was carried out in a semi-batch mode: batch charging of vegetable matter and continuous flow solvent. The laurel volatile oil was obtained working at 90 bar and 40°C in the extraction vessel, at 90 bar and –10°C in the first separator and at 20 bar and 15°C in the second one. The extraction of the fixed oil was run on the same samples of laurel previously treated at 90 bar; the laurel fixed oil was obtained working at 250 bar and 40°C in the extraction vessel and by using only one separator (at 20 bar and 15°C ) to recover the extract.

### Essential oil

### GC-MS Analysis

An Agilent Technologies Inc. (Santa Clara, CA, USA) model 6890N gas chromatograph was employed for analysis of the essential oils. It was equipped with a split-splitless injector, an autosampler Agilent model 7683 and an Agilent HP5 fused silica column; (5% phenyl-methylpolysiloxane, 30 m × 0.25 mm i.d., film thickness 0.25 μm). GC conditions used were: programmed heating from 60 to 280°C at 3°C/min followed by 30 min under isothermal conditions. The injector was maintained at 250°C. Helium was the carrier gas at 1.0 mL/min; the sample (1 μL) was injected in the split mode (1:20). The GC was fitted with a quadrupole mass spectrometer, MS, Agilent model 5973 detector. MS conditions were as follows: ionization energy 70 eV, electronic impact ion source temperature 200 °C, quadrupole temperature 100 °C, scan rate 1.6 scan/sec, mass range 50-500 u. Software adopted to handle mass spectra and chromatograms was a ChemStation. NIST 02 [18] and LIBR (TP) [19] Mass Spectra Libraries were used as references. Samples were run in chloroform with a dilution ratio of 1:100. Compounds were identified by matching their mass spectra and retention indexs with those reported in the literature [19]. Moreover, whenever possible, identification has been confirmed by injection of autentic sample of the compound. A quantitative analysis of each oil component (expressed in percentages) was carried out by peak area normalization measurement. The response factors were estimates using standard compounds having the same molecular weight of the compound families that constitute the essential oil (hydrocarbon monoterpenes, oxygenated monoterpenes, hydrocarbon sesquiterpenes and oxygenated sesquitepenes). Table 1 list the oil composition as % chromatographic peak areas.

### Fixed oil

### Preparation of fatty acids

Separation of fatty acids and α-tocopherol was obtained by mild saponification [20] as follows: the fixed oil (3 mg) was dissolved in EtOH (5 mL) and Desferal solution (25 mg/mL of H_2_O, 100 μL), an aqueous solution of ascorbic acid (25% w/v, 1 mL), and 10 N KOH (0.5 mL) were added. The mixtures were left in the dark at room temperature for 14 h. After addition of *n*-hexane (10 mL) and H_2_O (7 mL), samples were centrifuged for 1 h at 900*g*. The hexane phase with vitamin E was collected, the solvent was evaporated, the residue was dissolved in MeOH (500 μL) and aliquots of the samples were injected into the HPLC system. After addition of further *n*-hexane (10 mL) to the mixtures, samples were acidified with 37% HCl to pH 3-4 and then centrifuged for 1 h at 900*g*. The hexane phase with free fatty acids and hydroperoxides was collected and the solvent was evaporated. A portion of the dried residue was dissolved in CH_3_CN (500 μL) containing 0.14% CH_3_COOH (v/v) and aliquots of the samples were injected into the HPLC system. 

Aliquots of dried fatty acids were methylated with 14% BF_3_ in MeOH (1 mL) [21] for 30 min at room temperature. After addition of *n*-hexane (4 mL) and H_2_O (2 mL), samples were centrifuged for 20 min at 900*g*. The hexane phase with fatty acids methyl esters was collected, the solvent was evaporated, the residue was dissolved in *n*-hexane (100 μL) and aliquots of the samples were injected into the GC system. The recovery of fatty acids during saponification was calculated by using an external standard mixture. All solvents evaporation was performed under vacuum.

### HPLC analysis

Analysis of unsaturated fatty acids was carried out with an Agilent Technologies 1100 liquid chromatograph. Analyses of unsaturated fatty acids and conjugated dienes fatty acids hydroperoxides (HP) [20], detected at 200 and 234 nm respectively, were carried out with a Chrompack column, Inertsil 5 ODS-2 (150 mm × 4.6 mm, 5 μm particle size) with a mobile phase of CH_3_CN/H_2_O/CH_3_COOH (70/30/0.12, v/v/v) at a flow rate of 1.5 mL/min. The identification of the peaks was made using standard compounds and second derivative as well as conventional UV spectra, generated using the Agilent Chemstation A.10.02 software. The amount of α-tocopherol was measured by electrochemical detection [20], using a Thermo Separation Products (Milan, Italy) P1000 pump equipped with an electrochemical detector INTRO (Antec Leyden, Leiden, The Netherlands). An automatic injector, Triathlon (Spark Holland BV, AJ Emmen, The Netherlands) was also used. A C-18 Hewlett Packard ODS Hypersil column, 5 μm particle size, 100 × 2.1 mm, was used with a mobile phase of MeOH/CH_3_COONa 0.05M pH 5.5 (95/5, v/v) at a flow rate of 0.3 mL/min. Electrochemical detector was set at an oxidizing potential of 0.6 V. Data were collected and analysed using the Agilent Chemstation A.10.02. software.

### GC analysis

Fatty acid methyl esters were measured on a Hewlett-Packard HP-6890 gas chromatograph (Hewlett-Packard, Palo Alto, USA) with equipped with a flame ionisation detector and a cyanopropyl methylpolysiloxane HP-23 FAME column (30 m × 0.32 mm × 0.25 μm, Hewlett-Packard). Nitrogen was used as carrier gas at a flow rate of 2 mL/min. The oven temperature was programmed from 45 °C to 175 °C at a rate of 80 °C/min and held for 45 min; injector temperature was set at 250 °C, and detector temperature at 300 °C. The fatty acids methyl esters were identified by comparing the retention times with those of standard compounds. The percentage composition of individual fatty acids were calculated using a calibration curve with components injected at different concentrations, using the Hewlett-Packard A.05.02 software. 
